# Draft Genome Sequence of Bacillus albus *Strain* IB84, Isolated from Mexican Soil

**DOI:** 10.1128/MRA.00274-21

**Published:** 2021-04-29

**Authors:** Jorge H. García-García, Carlos J. Moreno-Peña, Cesar I. Hernández-Vásquez, Luis J. Galán-Wong, Benito Pereyra-Alférez

**Affiliations:** a Instituto de Biotecnología, Facultad de Ciencias Biológicas, Universidad Autónoma de Nuevo León, San Nicolas de los Garza, Nuevo León, Mexico; University of Rochester School of Medicine and Dentistry

## Abstract

Bacillus albus is a new species, but it lies on the borderline with Bacillus thuringiensis. In this work, we report a strain previously identified as Bacillus thuringiensis IB84, which now, based on average nucleotide identity and rRNA 16S, *gyrB*, *groEL*, and *xre* gene sequences, must be identified as Bacillus albus.

## Abstract (precis)

Bacillus albus is a new species, but it lies on the borderline with Bacillus thuringiensis. In this work, we report a strain previously identified as Bacillus thuringiensis IB84, which now, based on average nucleotide identity and rRNA 16S, *gyr*B, *gro*EL, and XRE gene sequences, must be identified as Bacillus albus.

## ANNOUNCEMENT

The IB84 strain was originally recognized as Bacillus thuringiensis because it is Gram positive and sporulated and produces a small parasporal crystal. This bacterium was originally isolated from agricultural soil collected in the state of San Luis Potosí, Mexico, using the methodology described by Travers et al. (1), and it belongs to the B. thuringiensis collection of the Instituto de Biotecnología of the Facultad de Ciencias Biológicas-UANL. Preliminary bioassays revealed that crystal inclusion proteins were not toxic against several insects (2).

For the whole-genome sequencing of Bacillus albus IB84, cells obtained from our laboratory cryostock were grown in 25 ml of LB broth for 6 h at 30°C, and then the pellet was recovered after centrifugation and washed with sterile water. The pellet was shipped under cold conditions to Genewiz (New Jersey, USA), where total DNA extraction was performed using the EasyPure genomic DNA kit (Genewiz), and the DNA library was prepared with the DNA prep (M) tagmentation, and IDT for Illumina DNA/RNA indexes set A tagmentation kits (Illumina, Inc., California, USA). The whole genome was sequenced using paired-end Illumina MiSeq sequencing, which resulted in 10,065,062 paired-end reads of 118 to 150 bp with a coverage of 250×. For genome assembly and annotation, we used Galaxy at http://usegalaxy.org (3). In brief, the sequence quality was determined with FastQC (Galaxy version 0.72+galaxy1) (4). Then, low-quality sequences and adapters were removed with Trimmomatic (Galaxy version 0.038.0) (5). Processed sequence reads were assembled with the Shovill pipeline, which includes the SPAdes assembler (Galaxy version 1.1.0+galaxy0) (6); the resulting contigs were annotated with the NCBI Prokaryotic Genome Annotation Pipeline (PGAP) using the best-placed reference protein set method (GeneMarkS-2+) (7, 8); 92.03% of all sequence reads were assembled in 156 contigs. Default parameters were used for all software.

We identified a 5.96-Mb genome with an *N*_50_ value of 155,983 bp, 6,015 open reading frames (ORF), 35.06% GC content, 74 tRNA operons, 6 rRNAs, and 5 noncoding RNAs (ncRNAs). The average nucleotide identity (ANI) of the whole genome determined during the GenBank submission process (9) showed 97.907% identity with the type genome of Bacillus albus; this and the sequence identities of rRNA 16S, *gyrB*, *groEL*, and *xre* genes suggest that this isolate belongs to the Bacillus albus species (10). This species has been described recently as having an important salt tolerance and the ability to degrade poultry waste processing industrial (PWPI) effluent (10, 11). The IB84 whole-genome analysis revealed sequences associated with assimilation and detoxification of nitrogen and arsenate compounds, as well as salt tolerance, antibiotic resistance, and oxidative stress genes.

The whole-genome sequence, assembly, and annotation of IB84 help to further understand *B. albus* and its evolutionary relationships with the *Bacillus* group. Furthermore, it allows us to study the possible advantages of its genetics, its biochemical machinery, and its biotechnological potential.

### Data availability.

The Bacillus albus IB84 whole-genome shotgun sequence has been deposited in GenBank under the accession number JADNYT000000000 and BioProject number PRJNA676033. The raw sequence files can be found under SRA number SRP301583.
